# Supplementary material for: Predicting innovative firms using web mining and deep learning
Source: PLoS One. 2021 Apr 1;16(4):e0249071. doi: 10.1371/journal.pone.0249071 (PMC8016297; doi:10.1371/journal.pone.0249071)
Supplement: Attachment — Submitted filename: Response.pdf [file pone.0249071.s001.pdf]

Dear Reviewer,

Thank you very much for the positive review. The author team has made every effort to improve all the formal errors and ambiguities in our manuscript that you mentioned. We sincerely hope that our revised manuscript will meet your requirements.

Comment 1: The authors wrote that the model under-estimate the share of product innovators in sectors for which a reference value is available but the share of product innovators for which no training data is available can be considered to be reasonable. It would be reasonable to think that a model's performance is better for the sector with a reference value than the sector without a reference value. If the converse is true, the less you have data with a reference value, the better the model's performance would be. The authors need to explain the sectoral performance of model in a different way.

Response 1: We fully agree with this keen observation. It is true that we are causing confusion for the reader here through an unclear choice of words. We have adjusted the corresponding passage in the manuscript. The corresponding passage has been marked in red in the manuscript. We hope that the new wording allows a clearer interpretation of our results.

Sincerely yours,

the team of authors
